# Addressing psychological resilience and its determinants among university students during the COVID-19 pandemic: a three-wave longitudinal study in Shandong Province, China

**DOI:** 10.1186/s12888-024-06175-3

**Published:** 2024-11-19

**Authors:** Lutong Pan, Jingjing Zhao, Mingli Pang, Jieru Wang, Yue Zhou, Rui Chen, Hui Liu, Xixing Xu, Baochen Su, Limei Nie, Jiajia Zhao, Shixue Li, Jiajia Li, Hexian Li, Fanlei Kong

**Affiliations:** 1 Centre for Health Management and Policy Research, School of Public Health, Cheeloo College of Medicine, Shandong University, Jinan, 250012 China; 2 NHC Key Lab of Health Economics and Policy Research, Shandong University, Jinan, 250012 China; 3 Institute of Health and Elderly Care, Shandong University, Jinan, 250012 China; 4 School of Foreign Languages and Literature, Shandong University, Jinan, 250100 China; 5 Department of Mathematics, College of Art and Science, New York University, New York, 10003 USA

**Keywords:** Psychological resilience, Mental health, Depression, Anxiety, Social mentality, University students, Longitudinal study, COVID-19

## Abstract

**Background:**

The relationship between public health emergencies and psychological distress had been well known, yet none research had been conducted on the trend in psychological resilience and its longitudinal determinants during the pandemic. This study aimed to explore the changes of psychological resilience of university students during COVID-19 pandemic, and further clarify the longitudinal relationship between family factors, mental health, social mentality and psychological resilience.

**Methods:**

Questionnaires were distributed to students from five universities in Shandong Province, China during the COVID-19. A total of 1635 students were finally included in this three-wave follow-up study using stratified random sampling method. Mental health was assessed by Depression Anxiety Stress Scale, social mentality was measured by the Bi-Dimensional Structure Questionnaire of Social Mentality, psychological resilience was evaluated by the Chinese version of the Psychological Resilience Scale. Repeated-measures analysis of variance was used to analyze the longitudinal changes of psychological resilience, generalized estimating equation (GEE) was conducted to estimate the determinants of psychological resilience.

**Results:**

Psychological resilience changed from 28.37 in Wave 1, 29.10 in Wave 2, and 29.15 in Wave 3 among the university students. The students who majored in Art (β = 0.872, *P* = 0.032), parents (mother β = 0.546, *P* = 0.035; father β = 0.718, *P* = 0.012) had a greater influence on children’s personality, and positive social mentality (β = 5.725, *P* < 0.001) were more likely to report a higher psychological resilience. Being female (β=-0.932, *P* < 0.001), not being a student leader (β=-0.911, *P* < 0.001), being anxious (β=-1.845, *P* < 0.001) and depressed (β=-1.846, *P* < 0.001), and negative social mentality (β=-0.803, *P* < 0.001) were less likely to report a higher psychological resilience.

**Conclusions:**

The psychological resilience of the university students in Shandong Province, China increased significantly from Wave 1 to Wave 3 during the COVID-19 pandemic. Majoring in Art, parents having a greater influence on children’s personality, better mental health, positive social mentality were more likely to report a higher psychological resilience, while female, not student leader, worse mental health, and negative social mentality were less likely to report a higher the psychological resilience.

## Introduction

The 2019 coronavirus disease (COVID-19) pandemic, was declared as an international public health emergency by the World Health Organization (WHO) on January 1st, 2020 [1], leading to substantial psychological distress [2]. University students, a group known for their sensitivity to public health emergencies [3], have reported increased anxiety, fear, and worry during the COVID-19 [4]. Psychological resilience is considered as a good adaptive potential of human beings when facing difficulties and adversities [5], which was related to mental health status of students [6, 7]. Assessing the psychological resilience could enhance the strategic allocation of resources and guide interventions aimed at mitigating the immediate effects of the pandemic on mental health [8]. One study conducted among the U.S. adults during the COVID-19 pandemic showed that the resilience level was lower than the published norms [9]. Meanwhile, some research also demonstrated that traumatic experiences could stimulate the positive responses and promote the resilience level [10]. Given the unique challenges faced by university students during the COVID-19 pandemic, and the uncertain impact of adverse life experience on the resilience, it is critical to investigate the changes of psychological resilience and their determinants to develop the tailored interventions on enhancing the university students’ wellbeing.

Family is the foundational unit of a society [11] and existed study have found the family environment and family cohesion could enhance individual’s resilience [12]. This foundational resilience is further reinforced by the family’s ongoing role as a protective resource, as demonstrated by Dong et al. [13]. The style of parenting has been closely linked to psychological resilience [14], for instance, a secure relationship with parents could positively affect resilience [15, 16]. Additionally, studies have shown that parenting styles could have either a positive or negative influence on the resilience of children [17, 18]. Southwick et al. further emphasized the significant impact of the linkages between individuals, families, and communities on individual resilience [19]. One qualitative study among the young adult children of parents found that family could exert a significant influence on the children’s resilience [20]. However, none study had clarified the longitudinal relationship between the family factors and resilience among the university students during the COVID-19 pandemic.

Increasing levels of stress, anxiety, and depression were also reported among the child and adolescent during the COVID-19 pandemic [21]. Mesman et al. emphasized the strong association between resilience and mental health in children and adolescents, suggesting that resilience deserved a more prominent role in research [22]. A study found that children and adolescents in Germany felt significantly burdened by measures (such as confinement, social alienation, and home schooling) could lead to a significant increase in their mental health problems [23]. Some cross-sectional studies have provided substantial evidence of a negative association between resilience and depression [24–26], as well as a negative association between resilience and anxiety in the general Australian public [27]. However, none research had ever explored the longitudinal changes of resilience and its relationship with mental health among the university students during the COVID-19 pandemic.

Social mentality refers to the cognitions, emotions, values, and behaviors that permeate society as a whole or social groups or categories over a period of time [28]. It is widely acknowledged that social mentality is macroscopic and dynamic, formed under the influence of the social environment and culture in a certain period of time, closely related to social changes, and transcends individual psychological social psychological phenomena [29]. Numerous studies identified a significant positive correlation between psychological resilience and optimistic tendencies among the university students, as well as a significant negative correlation with pessimistic tendencies [30]. Individuals with a more positive mentality tend to exhibit higher levels of psychological resilience [31], while the shifts in social mentality reflect the pandemic’s profound impact on society and the collective resilience of individuals at a group level [32, 33]. To date, none research had explored the longitudinal association between the social mentality and psychological resilience among the university students during the COVID-19 pandemic.

The majority of existed studies on the psychological resilience of the university students during COVID-19 mainly used the cross-sectional designs [34, 35], which fall short in capturing the dynamics of resilience over time. Longitudinal studies, by contrast, are good at deepening our understanding [36] and examining the changes of research issues over time [37]. To date, few studies used the longitudinal designs, not mention the effect of family factors, mental health, social mentality upon the psychological resilience. Thus, this study aimed to explore the longitudinal changes of psychological resilience, as well as the relationship between family factors, mental health, social mentality and psychological resilience among university students in Shandong Province, China during COVID-19 pandemic.

## Method

### Study design and sampling method

This longitudinal study was conducted in five universities, in Shandong Province, China. A stratified random sampling method that considered geographical location (east, middle, or west) and college category (key or general) was used in this study. First, five universities were randomly selected from a list of institutions in Shandong Province, including Shandong University, Weifang University, Yantai University, Jining College, and Liaocheng University, ensuring a mix of geographical locations and academic statuses. Second, within each university, seven majors (including Engineering, Science, Agriculture, Literature, Liberal art, Art, and Medicine ) were randomly selected from the available options, ensuring diversity in academic disciplines. Third, students from one class were randomly chosen from each year of each selected major to join the survey.

The Wave 1 of this study was conducted when graduating students returned to campus on June 17 to 24, 2020, while 4,959 students (valid response rate: 85%) completed the survey. The Wave 2 occurred on November 1 to 6, 2020, when non-graduating students returned to campus after two months of the new semester started, with a total of 4,832 students (valid response rate: 82%) completed the survey. The Wave 3 occurred on January 18 to 25, 2021, when China’s government began to provide COVID-19 vaccines to all citizens while students returned home after completing the autumn-term study on campus, with a total of 4,408 students (valid response rate: 91.54%) completed the survey. By using the student ID numbers to match these three Waves’ responses, a total of 1,635 university students were found to have participated in this longitudinal survey and selected as our study participants.

### Quality control

The full-time tutors of the university students organized the process of distributing the online questionnaires, which were sent via WeChat (similar to Facebook, one popular social network App in China) to potential respondents in the sampled classes, asking students to fill in the questionnaires within a set time frame. One IP address was only allowed to fill in the questionnaire one time to improve the quality of the data collection. The questionnaires were tracked by student ID number and telephone number to minimize the number of missed responses. A pilot study was conducted to refine the questionnaires before the Wave 1 questionnaires survey. To control the quality of the questionnaires, a trap question was strategically placed within each questionnaire (such as ‘this question was used to check the level of careful reading, please select ‘least likely’’) to assess the respondents’ level of attention and engagement. Any answer to this question that deviated from the prescribed ‘least likely’ option was deemed illogical.

### Inclusion and exclusion criteria

Inclusion in the study was determined by the respondents being full-time undergraduate students enrolled in the selected majors, who voluntarily agreed to participate. The exclusion criteria were applied to ensure the quality and validity of the collected data. The results of each Wave were screened to exclude those that did not meet the following criteria: (1) incorrect answers to the trap questions, (2) socio-demographic characteristics that were missing or not relevant to the survey, and (3) responses that were not logically consistent with other related responses.

### Sample characteristics

**Fig. 1:**
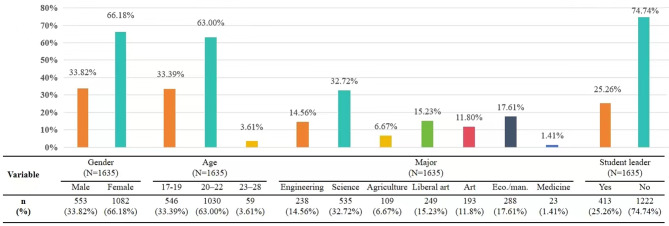
Socio-demographic characteristics of participants in three waves (*N* = 1635). *Note*: Eco/man: Economic management

Displays of the participants’ socio-demographic characteristics for 1635 participants could be found in Fig. 1. The sample for these longitudinal analyses were 1635 participants (66.18% females, 33.82% males) from five different universities in Shandong Province who had participated in all three waves questionnaire survey. In terms of age distribution, the participants were divided into three groups: 546 individuals aged 17–19 (33.39%), 1030 (63.00%) aged 20–22, and 59 (3.61%) aged 23–28. Concerning the seven disciplines (Engineering, Science, Agriculture, Literature, Liberal art, Art, and Medicine), students who were studying science accounted for 32.7%, while 1.4% of them were studying medicine. Approximately one-fourth (25.3%) of the respondents were student leaders.

### Measurement

#### Socio-demographic characteristics

Socio-demographic characteristics included gender (male vs. female), age (17–19, 20–22, or 23–28), major (science, engineering, agriculture, economic management, liberal art, art, or medicine), student leader (yes or no).

#### Family factors

Family factors included four parts: family composition, economic status, educational style, and parental influence on children. For family composition, whether they were surrounded (family members, neighbors, or relatives) by medical workers were asked; and for parental influence on children, which parent (father or mother) had a greater influence on children’s personality were asked.

#### Depression anxiety stress scale (DASS-21)

The DASS-21 was used to assess participants’ depression, anxiety, and stress. The scale contains seven items on each dimension (depression, anxiety and stress). Responses ranged from 0 (*did not apply to me at all*) to 3 (*applied to me very much*) [38].

As the DASS-21 is a short form version of the DASS-42, DASS-21 scores were multiplied by 2 to characterize the level of severity relative to the population. Depression level was classified into five categories: normal (0–9), mild (10–13), moderate (14–20), severe (21–27), and very severe (28+). Similarly, for anxiety, categories were normal (0–7), mild (8– 9), moderate (10–14), severe (15–19), and very severe (20+). Stress scores range from normal (0–14), mild (15–18), moderate (19–25), severe (26–33), to very severe (34+) [39]. Specifically, students falling in the moderately, severely, and extremely severely depressed, anxious, and stressed categories were considered to be depressed, anxious, and stressed, respectively; others were considered to be not depressed, not anxious, and not stressed, respectively [40]. Cronbach’s alphas in the current study for the depression, anxiety, and stress subscales were 0.901, 0.859, and 0.871 respectively at Wave 1, 0.915, 0.882, and 0.895 respectively at Wave 2, and 0.927, 0.896, and 0.914 respectively at Wave 3, indicating acceptable reliability of the DASS-21 Scale in this study.

#### The bi-dimensional structure questionnaire of social mentality (B-DSMQ)

To assess social mentality, the Bi-Dimensional Structure Questionnaire of Social Mentality (B-DSMQ) were used. The scale is divided into two dimensions, one for ‘individual-public’ and the other for ‘positive-negative’. The former is the subject dimension of social mentality, which includes both micro and macro perspectives; the latter is the valence dimension of social mentality, which includes both negative and positive perspectives. The scale includes 46 words or phrases (21 for positive social mentality and 25 for negative social mentality). Meanwhile, 25 of the above phrases were used for the individual mind and 34 for the public mind. The B-DSMQ includes positive individual mentality (PIM), negative individual mentality (NIM), positive public mentality (PPbM), negative public mentality (NPbM), positive social mentality (PSM), negative social mentality (NSM), and balanced social mentality (BSM). PSM is comprised of PIM and PPbM, while NSM is comprised of NIM and NPbM. BSM is the balance between PSM and NSM (calculated by ‘PSM-NSM’), indicating the proportion of positive social mentality and describing overall social mentality based on subject (individual-public) and valence (positive-negative). The scale used the PANAS (Positive and Negative Affect Schedule, a commonly used scale evaluating emotions) item presentation method, displaying specific phrases of social mentality, such as being insecure, being hopeful, being faithful, being tolerant, being harmonious, being unfair, being honest, being grateful, and being supportive. Participants were asked to rate the items both personally and from the viewpoint of the general public. On a 6-point Likert measure, responses ranged from 1 (not at all) to 6 (mostly). Higher BSM score suggests a more positive overall social mentality. Previous research had verified the validity and reliability of the B-DSMQ (Cronbach’s alpha ranged from 0.917 to 0.970) [41]. In this research, the Cronbach’s alpha of B-DSMQ ranged from 0.946 to 0.975 at Wave 1, 0.942 to 0.981 at Wave 2, and 0.944 to 0.981 at Wave 3, demonstrating acceptable reliability scores.

#### Psychological resilience (CD-RISC-10)

A simplified 10-item version of the CD-RISC-10 was used to evaluate the participants’ ability to return to a normal state when encountering dilemmas or challenges [42, 43]. This scale has demonstrated excellent validity and reliability among the Chinese people (Cronbach’s alpha = 0.969) [44]. For this assessment, a five-point Likert scale was employed, where 0 meant not true at all, 1 meant rarely true, 2 meant sometimes true, 3 meant often true, and 4 meant true nearly all the time. As a sum of all the items’ points, participants’ total scores ranged from 0 to 40 points. A higher total score denoted a higher level of resilience. In the current study, the reliability of the CD-RISC-10 also demonstrated acceptable values: Cronbach’s alpha for the scale was 0.968, 0.973, and 0.979 in Wave 1, Wave 2, and Wave 3.

### Statistical analysis

First, descriptive analysis was conducted to display the characteristics of participants; repeated-measures analysis of variance (RM-ANOVA) was performed to test if the mental health, social mentality and psychological resilience varied with time; pairwise comparisons analysis was employed to show the differences of psychological resilience in Waves 1–3. The null hypotheses of the tests were the mental health, social mentality and psychological resilience did not change with time (or different wave). The *P*-values of the hypothesis tests were then adjusted by Bonferroni-Holm correction. Second, the longitudinal associations between demographic characteristics, family factors, mental health, social mentality, and psychological resilience were analyzed using generalized estimating equations (GEEs), and four models were built to control for confounding variables. Demographic characteristics were included in Model 1; demographic characteristics and family factors were included in Model 2; demographic characteristics, family factors, and mental health were included in Model 3; demographic characteristics, family factors, mental health and social mentality were included in Model 4. All statistical tests were performed using IBM SPSS 25.0. Statistical significance was set at *P* < 0.05.

### Institutional review board statement

The study was designed in accordance with the tenets of the Declaration of Helsinki, 1996 and was approved by the ethical committee of Shandong University before data collection (Task No. LL20200201).

## Results

### Distribution of family factors

Table 1 presented family-related characteristics among 1,635 participants across three survey waves. The majority of participants (83.43%) were not surrounded by medical workers, while 16.57% were. In terms of family financial status, the majority (56.51%) reported average financial conditions, with 36.64% classified as poor and 5.63% as extremely poor; only 1.22% were considered rich. The predominant parenting style was the democratic deliberative type, accounting for 85.87% of responses, followed by strict control (7.83%), doting and pampering (1.65%), and hands-off (4.65%). Regarding parental influence on children’s personality, 46.36% of participants identified their mother as having a greater impact, 28.07% identified their father, and 25.57% reported that it was unclear.

**Table 1:** Distribution of family factors of participants in three waves (*N* = 1635)

Variable	Classification	Total *n* (%)
Surrounded (family members, neighbors, relative) by medical workers	Yes	271 (16.57%)
	No	1364 (83.43%)
Family’s financial situation	Extremely poor	92 (5.63%)
	Poor	599 (36.64%)
	Average	924 (56.51%)
	Rich	20 (1.22%)
The way parents educate their children	Strict control type	128 (7.83%)
	Hands-off type	76 (4.65%)
	Democratic deliberative type	1404 (85.87%)
	Doting and pampering type	27 (1.65%)
Which parent had a greater influence on children’s personality	Unclear	418 (25.57%)
	Father	459 (28.07%)
	Mother	758 (46.36%)

### The longitudinal changes of depression, anxiety, stress, social mentality, and psychological resilience in three waves among the university students in Shandong Province, China

**Fig. 2:**
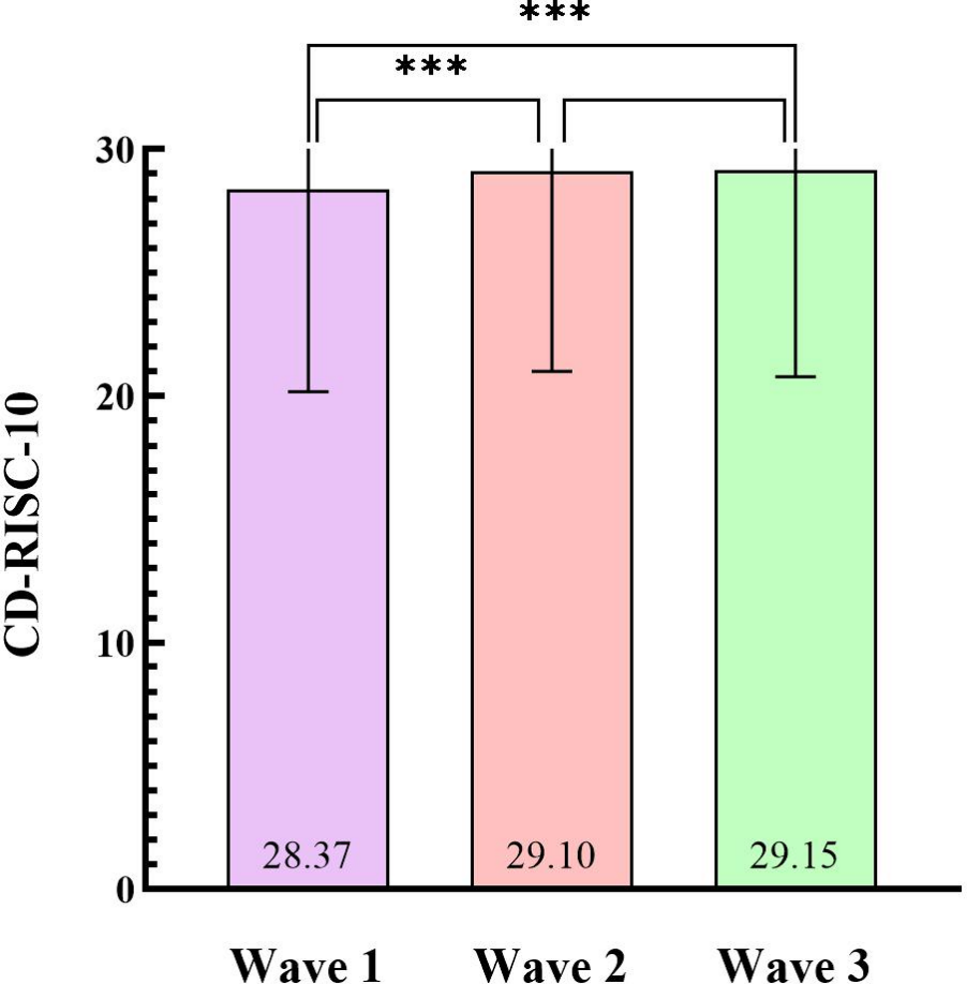
Trends of CD-RISC-10 (Conner-Davidson resilience scale) in three waves. *Notes*: ***: *P* < 0.001 and adjusted *P* < 0.05 after Bonferroni correction. CD-RISC-10, Conner-Davidson Resilience Scale

The longitudinal changes of the participants’ depression, anxiety, stress, various dimensions of social mentality and psychological resilience in three waves were shown in Table 2. Concerning the mental health, the score of stress decreased significantly (*P* < 0.001) from Wave 1 (9.34 ± 8.11) to Wave 3 (8.27 ± 8.39), the score of depression also decreased from Wave 1 (7.56 ± 7.88) to Wave 3 (7.48 ± 8.36), while the score of anxiety increased from Wave 1 (7.22 ± 7.43) to Wave 3 (7.27 ± 7.82). As for the social mentality, the average score of PIM and BSM increased significantly (*P* < 0.001) from Wave 1 to Wave 3, while the average score of PPbM, NIM, NPbM, NSM and PSM decreased significantly (*P* < 0.001) from Wave 1 to Wave 3. Regarding the psychological resilience (as shown in Fig. 2), the score of CD-RISC-10 increased significantly from Wave 1 (28.37 ± 8.20) to Wave 2 (29.10 ± 8.09; *P* < 0.001) and Wave 1 (28.37 ± 8.20) to Wave 3 (29.15 ± 8.38; *P* < 0.001).

**Table 2:** Longitudinal changes of university students’ depression, anxiety, stress, various dimensions of social mentality, and psychological resilience (mean ± SD)

Variables	Wave1 Jun. 2020	Wave2 Nov. 2020	Wave3 Jan. 2021	*P*
Depression	7.56 ± 7.88	7.51 ± 8.09	7.48 ± 8.36	0.924
Anxiety	7.22 ± 7.43	7.52 ± 7.67	7.27 ± 7.82	0.203
Stress	9.34 ± 8.11	9.03 ± 8.31	8.27 ± 8.39	<0.001*
PIM	4.42 ± 1.00	4.41 ± 0.77	4.67 ± 0.93	<0.001*
PPbM	4.52 ± 0.99	4.09 ± 0.59	4.10 ± 0.60	<0.001*
NIM	2.12 ± 0.96	2.20 ± 0.84	2.02 ± 0.94	<0.001*
NPbM	2.28 ± 1.01	2.06 ± 0.91	2.07 ± 0.92	<0.001*
PSM	4.48 ± 0.95	4.22 ± 0.62	4.33 ± 0.70	<0.001*
NSM	2.21 ± 0.93	2.12 ± 0.83	2.05 ± 0.89	<0.001*
BSM	2.27 ± 1.63	2.10 ± 1.27	2.28 ± 1.43	<0.001*
CD-RISC-10	28.37 ± 8.20	29.10 ± 8.09	29.15 ± 8.38	<0.001*

*Notes*: Data are shown as mean ± SD. *: Adjusted *P* < 0.05 after Bonferroni correction. SD indicates standard deviation; PIM, positive individual mentality; NIM, negative individual mentality; PPbM, positive public mentality; NPbM, negative public mentality; PSM, positive social mentality; NSM, negative social mentality; BSM, balanced social mentality; CD-RISC-10, Conner-Davidson Resilience Scale; Repeated-Measures Analysis of variance was performed to explore the variance of mental health, social mentality and psychological resilience with time

### Association between socio-demographic characteristics, family factors, mental health, social mentality and psychological resilience

The findings of GEE analysis of psychological resilience were presented in Table 3. Socio-demographic characteristics related variables were introduced in Model 1, while Model 2 furtherly concluded family factors related variables based on Model 1, Model 3 further concluded mental health related variables based on Model 2, Model 4 further concluded social mentality related variables based on Model 3. The research results showed that there was significant correlation between socio-demographic characteristics, family factors, mental health, social mentality and psychological resilience.

Specifically, concerning the socio-demographic characteristic, the students who majored in Art were more likely to report a higher psychological resilience (β = 0.872, *P* = 0.032), while being female (β=-0.932, *P* < 0.001), not being a student leader (β=-0.911, *P* < 0.001) were found to be less likely to report a higher psychological resilience. Regarding the family factors, either mother (β = 0.546, *P* = 0.035) or father (β = 0.718, *P* = 0.012) had greater influence on children’s personality were more likely to report a higher psychological resilience than those who were unclear about it. As for the mental health, being anxious (β=-1.845, *P* < 0.001) and depressed (β=-1.846, *P* < 0.001) were less likely to report a higher psychological resilience. Additionally, positive social mentality was more likely to report a higher psychological resilience (β = 5.725, *P* < 0.001), while negative social mentality (β=-0.803, *P* < 0.001) were less likely to report a higher psychological resilience.

**Table 3:** Longitudinal associations of demographic characteristics, family factors, mental health, social mentality with psychological resilience based on GEE

Variables	Model 1 (Demography)				Model 2 (Model 1 + Family Factors)				Model 3 (Model 2 + DASS-21)				Model 4 (Model 3 + B-DSMQ)			
	β	SE	95% CI	*P*	β	SE	95% CI	*P*	β	SE	95% CI	*P*	β	SE	95% CI	*P*
Gender																
Female	0.073	0.394	-0.7,0.846	0.853	0.056	0.382	-0.693,0.806	0.883	-0.730	0.319	-1.355,-0.106	0.022	-0.932	0.244	-1.409,-0.455	< 0.001
Male	Ref.				Ref.				Ref.				Ref.			
Age																
23–28	0.232	0.960	-1.65,2.114	0.809	0.707	0.942	-1.138,2.552	0.453	0.846	0.723	-0.57,2.263	0.242	0.693	0.529	-0.345,1.73	0.191
20–22	0.151	0.367	-0.568,0.871	0.680	0.289	0.361	-0.419,0.998	0.423	0.365	0.303	-0.228,0.958	0.228	0.364	0.227	-0.081,0.808	0.109
17–19	Ref.				Ref.				Ref.				Ref.			
Major																
Economic management	-1.610	1.479	-4.509,1.289	0.276	-1.411	1.425	-4.203,1.381	0.322	-0.855	1.333	-3.466,1.757	0.521	0.200	0.812	-1.391,1.792	0.805
Art	-0.020	0.659	-1.312,1.273	0.976	-0.267	0.654	-1.549,1.016	0.683	0.125	0.540	-0.932,1.183	0.816	0.872	0.407	0.074,1.669	0.032
Liberal art	-0.272	0.720	-1.683,1.138	0.705	-0.476	0.716	-1.878,0.927	0.506	-0.220	0.596	-1.388,0.947	0.711	0.442	0.431	-0.402,1.286	0.304
Agriculture	-1.320	0.657	-2.607,-0.033	0.044	-1.548	0.642	-2.807,-0.289	0.016	-0.814	0.522	-1.837,0.209	0.119	-0.062	0.413	-0.871,0.747	0.880
Science	0.111	0.818	-1.491,1.714	0.892	-0.113	0.809	-1.699,1.473	0.889	-0.215	0.649	-1.487,1.057	0.741	-0.040	0.492	-1.004,0.923	0.935
Medicine	-0.550	0.568	-1.663,0.564	0.334	-0.662	0.564	-1.766,0.443	0.240	-0.427	0.464	-1.336,0.482	0.357	0.040	0.339	-0.623,0.704	0.905
Engineering	Ref.				Ref.				Ref.				Ref.			
Student leader																
No	-1.614	0.388	-2.374,-0.855	< 0.001	-1.375	0.385	-2.131,-0.62	< 0.001	-1.131	0.315	-1.748,-0.514	< 0.001	-0.911	0.238	-1.376,-0.445	< 0.001
Yes	Ref.				Ref.				Ref.				Ref.			
Surrounded (family members, neighbors, relative) by medical workers																
No					0.112	0.446	-0.761,0.986	0.801	-0.235	0.365	-0.951,0.481	0.520	-0.251	0.283	-0.806,0.304	0.375
Yes					Ref.				Ref.				Ref.			
Family’s financial situation																
Extremely poor					-1.063	2.125	-5.228,3.102	0.617	-0.344	1.756	-3.786,3.098	0.845	0.457	1.330	-2.149,3.064	0.731
Poor					-1.990	2.002	-5.914,1.934	0.320	-1.750	1.655	-4.995,1.494	0.290	-0.959	1.259	-3.427,1.509	0.446
Average					-1.019	1.994	-4.927,2.889	0.609	-1.029	1.647	-4.258,2.2	0.532	-0.619	1.254	-3.078,1.839	0.621
Rich					Ref.				Ref.				Ref.			
The way parents educate their children																
Doting and pampering type					-0.563	1.504	-3.511,2.385	0.708	0.603	1.295	-1.935,3.14	0.642	1.352	0.964	-0.536,3.241	0.161
Democratic deliberative type					2.385	0.695	1.024,3.746	0.001	1.011	0.608	-0.181,2.202	0.096	0.576	0.450	-0.306,1.458	0.200
Hands-off type					-0.022	0.955	-1.892,1.849	0.982	0.192	0.837	-1.448,1.831	0.819	0.389	0.590	-0.768,1.546	0.510
Strict control type					Ref.				Ref.				Ref.			
Which parent had a greater influence on children’s personality																
Mother					1.499	0.411	0.694,2.305	< 0.001	1.135	0.343	0.463,1.806	0.001	0.546	0.259	0.038,1.053	0.035
Father					2.354	0.455	1.463,3.244	< 0.001	1.648	0.382	0.9,2.396	< 0.001	0.718	0.287	0.156,1.28	0.012
Unclear					Ref.				Ref.							
Psychological distress:																
Stress																
Stressed									-0.846	0.378	-1.587,-0.105	0.025	-0.204	0.337	-0.864,0.456	0.545
Not stressed									Ref.				Ref.			
Anxiety																
Anxious									-4.004	0.311	-4.615,-3.394	< 0.001	-1.845	0.270	-2.375,-1.315	< 0.001
Not anxious									Ref.				Ref.			
Depression																
Depressed									-4.796	0.338	-5.458,-4.134	< 0.001	-1.846	0.306	-2.446,-1.247	< 0.001
Not depressed									Ref.				Ref.			
PSM													5.725	0.219	5.296,6.153	< 0.001
NSM													-0.803	0.189	-1.172,-0.433	< 0.001
BSM													Ref.			

*Note*: DASS-21, Depression Anxiety Stress Scale; B-DSMQ, The Bi-Dimensional Structure Questionnaire of Social Mentality; PSM, positive social mentality; NSM, negative social mentality; BSM, balanced social mentality

## Discussion

This study reported the changes of mental health, social mentality, and psychological resilience among 1,635 Chinese university students from June 2020 to January 2021 during the COVID-19 pandemic, as well as investigated the longitudinal associations between socio-demographics characteristics, family factors, mental health, social mentality, and psychological resilience. To our knowledge, this is the first study clarified the longitudinal changes of psychological resilience and its relationship with family factors, mental health and social mentality among the university students during the COVID-19 pandemic in Shandong Province, China.

In this longitudinal survey, psychological resilience increased significantly from Wave 1 to Wave 3 during the COVID-19 pandemic, which was consistent with Zhu, H., et al.’s research that found the psychological resilience level of relocated adolescents increased over time under the pandemic [45]. Notably, the significant increase in Wave 2 compared to Wave 1 (*P* < 0.001) may attributed to the re-establishment of regular campus life. Effective control measures made it possible for university students to return to school after a six-month period of staying at home [46]. This improving situation may boost students’ confidence, which could improve psychological resilience [47]. Moreover, the significant increase of the psychological resilience in Wave 3 compared to Wave 1 may contribute to the government’s vaccination program launched on December 2020 and the duration were fairly long between Wave 1 and Wave 3 (from 17 to 24 June 2020 to 18–25 January 2021). The none-significant difference between Wave 2 and Wave 3 may be due to the fairly short duration between these two surveys, although the government’s vaccination program were also launched between them.

The study found that female students were less likely to report a higher psychological resilience, which was in line with the previous researches [48, 49]. This may due to the female students’ increased susceptibility to negative emotions [50], and the profound psychological impact of the outbreak [51] leading to a reduced capacity for resilience [52]. Additionally, the research found that art students were more likely to exhibit a higher psychological resilience, which was similar to the previous study [53] and shown that arts exposure was associated with positive psychological constructs [54], including psychological resilience [55]. This relationship is further supported by studies indicating that art education can play a significant role in fostering resilience [56, 57]. Furthermore, student leaders were found to be more likely to exhibit higher psychological resilience, which was consistent with the research showed student leaders had higher psychological resilience scores than non-leaders [58]. This may be explained by the fact that dealing with student affairs enhanced the students leaders’ sense of empowerment and self-esteem, which was positively correlated with psychological resilience [59]. Moreover, student leaders often engage more actively in extracurricular activities and social interactions, which can further cultivate resilience [60].

The study revealed that students who reported a greater influence from either their father or mother were more likely to have higher psychological resilience, which was consistent with previous studies demonstrating that father involvement had a direct effect on enhancing adolescent resilience [61] and strong mother–child relationships played a crucial role in resilience development [62]. For instance, a study by Feldman [63] discussed the multifaceted contributions of fathers to child resilience, including promoting adaptation to social rules and providing a material background that fosters resilience. Similarly, research by Fenning et al. [64] examines how mother-child interaction can predict reduced likelihood of intellectual disability, underscoring the importance of early nurturing environments in shaping resilience. As Luthara and Brownb pointed out, relationships between family members were the ‘root’ of resilience: the presence of support, love and security nurtures resilience by harnessing individuals’ inherent strengths [65]. In the Chinese context, when students were more closely connected to their parents, strong family connection may contribute to their life satisfaction in shaping their resilience [66].

The findings showed that being anxious and depressed were less likely to report a higher psychological resilience, which was consistent with previous studies’ which found higher levels of mental health symptoms among youth could predict lower levels of resilience [67]. Both depressive [68, 69] and anxiety [70] symptoms could interfere with their coping capacities, and were clarified to have lower psychological resilience. Moreover, a three-wave cross-lagged study has revealed a reciprocal relationship between resilience and both depression and anxiety symptoms, suggesting that while resilience could ameliorate mental health problems, these problems may also negatively impact resilience [71]. This dynamic relationship underscores the complexity of resilience and the importance of considering the broader mental health context when examining this construct.

In line with previous research [72], positive social mentality was found to be more likely to report a higher psychological resilience, while negative social mentality was less likely to report a higher psychological resilience. More and more researches found that positive social processes may promote more positive and adaptive coping in the management of traumatic experiences [73]. This aligned with the findings that highlight the role of social support and a sense of community in building resilience [74]. Studies have shown that positive societal measures [75] and a favorable social climate [76] could enhance the students’ psychological resilience.

### Implications

Based on the results above, the implication could be as follow. First, more attention should be paid to the female students since they are the vulnerable group on the lower psychological resilience. Second, fostering a good family atmosphere as well as strengthening family support and connections for university students were advocated since the family was a primary source of psychological resilience [13, 77] and a positive force to enhance the individual’s resilience [78, 79]. Third, it’s also recommended to shape a healthy social mentality by group interventions and guidance, promoting public health education, and creating a positive social climate to enhance psychological resilience [80]. Furthermore, in light of the COVID-19 pandemic, it is crucial to enhance public health education to ensure students are well-informed about prevention measures and the importance of mental health during these challenging times. Finally, the universities could provide appropriate mental health services for students to enhance their psychological resilience.

### Limitations

First, self-reported data was used in this study, which may increase the possibility of recall bias. Second, some respondents could not participate in three waves due to graduation, preparation for final exams, internship, and absence from school, which made the number of sample lower than expected. Third, the findings from the university students may not be applicable to the general population, especially those with lower levels of education. Forth, the study’s scope was limited in Shandong Province, which may not represent the educational context of the entire country. Additionally, the study was only conducted during the COVID-19 pandemic. The absence of pre-COVID data may limit our ability to conduct more comparisons and find more useful results.

### Strengths

Despite these limitations, several strengths distinguish our study. First, our study was a longitudinal design, which could track psychological resilience trends over the pandemic’s course. Second, our large sample size from five diverse universities could enhance the findings’ generalizability. Third, the utilization of comprehensive and validated instruments, such as the Depression Anxiety Stress Scale, the Bi-Dimensional Structure Questionnaire of Social Mentality, and the Chinese version of the Psychological Resilience Scale could improve the study’s methodological rigor.

## Conclusion

The psychological resilience of the university students in Shandong Province, China increased significantly from Wave 1 to Wave 3 during the COVID-19 pandemic. Majoring in Art, parents having a greater influence on children’s personality, better mental health, positive social mentality were more likely to report a higher psychological resilience, while female, not student leader, worse mental health, and negative social mentality were less likely to report a higher the psychological resilience. To enhance the psychological resilience of university students in the post-COVID-19 era, more attention should be paid to the vulnerable students, as well as offering a better family environment, providing more preventive individual mental health services and social mentality education.

## Data Availability

Under reasonable requirements, the data and material of this study can be obtained from the corresponding author. The data are not publicly available due to privacy restrictions.

## Abbreviations

DASS: 21 Depression Anxiety Stress Scale
B-DSMQ: Bi-Dimensional Structure Questionnaire of Social Mentality
CD-RISC: Chinese version of the Psychological Resilience Scale
RM-ANOVA: Repeated-Measures Analysis Of Variance
GEE: Generalized Estimating Equation
PIM: Positive Individual Mentality
NIM: Negative Individual Mentality
PPbM: Positive Public Mentality
NPbM: Negative Public Mentality
PSM: Positive Social Mentality
NSM: Negative Social Mentality
BSM: Balanced Social Mentality
CI: Confidence Interval
SD: Standard Deviation
